# Torsion of Tubo-Ovarian Complex during Pregnancy: A Case Report

**DOI:** 10.3390/medicina60091471

**Published:** 2024-09-09

**Authors:** Taruna Agrawal, Jhia Jiat Teh, Stergios Bobotis, Elisavet Arsenaki, Selim Maxwell, Konstantinos S. Kechagias, Suparna Sinha, Nosheen Rashid

**Affiliations:** 1 Department of Obstetrics and Gynaecology, The Hillingdon Hospitals NHS Foundation Trust, Uxbridge UB8 3NN, UK; jhia.teh@nhs.net (J.J.T.); konstantinos.kechagias18@imperial.ac.uk (K.S.K.); suparnasinha@nhs.net (S.S.); nosheen.rashid1@nhs.net (N.R.); 2 Department of Metabolism, Digestion and Reproduction, Imperial College London, London SW7 2AZ, UK; stergios.bobotis2@nhs.net (S.B.); e.arsenaki@nhs.net (E.A.); 3 Department of Accident & Emergency, Maidstone & Tunbridge Wells NHS Trust, Maidstone ME16 9QQ, UK; selim.maxwell1@nhs.net

**Keywords:** torsion, acute abdomen, high-risk pregnancy, surgical complications

## Abstract

*Introduction:* Adnexal torsion is characterised by the rotation of the ovary and, occasionally, the fallopian tube around their supporting ligaments by more than 45 degrees. It predominantly occurs during the first and second trimesters of pregnancy, with an incidence of up to 0.1% in the third trimester. Dermoid and functional ovarian cysts, most commonly associated with benign serous cystadenomas, are frequently identified among the torted adnexal masses. *Case Presentation:* We report the case of a 32-year-old primigravida with a known ovarian cyst diagnosed in the first trimester, which was managed conservatively throughout the pregnancy. At 36 weeks of gestation, she presented with abdominal pain and was subsequently managed with an emergency caesarean section at 37 weeks due to the development of an acute surgical abdomen. During the procedure, a torted left tubo-ovarian complex was excised, with partial preservation of the healthy-appearing ovarian tissue. Histopathological examination identified the mass as a benign serous cystadenoma. *Conclusions:* Ovarian torsion during pregnancy poses a significant diagnostic challenge. The decision between conservative management and surgical intervention is primarily guided by a high index of suspicion for torsion.

## 1. Introduction

Ovarian torsion is a critical gynaecological emergency characterised by the twisting of the ovary and, occasionally, the fallopian tube around their supporting ligaments. This torsion compromises the blood supply to the adnexa, leading to tissue ischaemia and potential ovarian necrosis. Rapid diagnosis and intervention are essential to prevent irreversible damage to the reproductive organs and to mitigate both local and systemic complications. Delayed treatment can result in permanent loss of ovarian function and broader health issues [1].

Ovarian torsion in pregnancy is uncommon, with diagnosis being particularly challenging due to several factors. The gravid uterus distorts normal abdominal anatomy, complicating physical examination. Additionally, the nonspecific symptoms associated with adnexal torsion overlap with a broad spectrum of other potential diagnoses, making accurate identification more difficult [2]. The estimated incidence of ovarian torsion in pregnancy is 1–5 in 10,000 pregnancies, although higher incidence rates have also been reported in the literature [3]. Right-sided torsions are more common than left-sided torsions because the sigmoid colon limits the mobility of the left ovary. Torsions are more commonly seen in the first and second trimester and rarely in the third trimester of pregnancy, and they are associated with high patient morbidity and foetal mortality if not immediately treated [4].

In this article, we present a rare case of tubo-ovarian complex torsion occurring in the third trimester of pregnancy, which escalated into an acute abdomen, necessitating an emergency caesarean section. We also review the existing literature on similar cases, emphasising the diagnostic challenges and management strategies related to this uncommon condition during pregnancy.

## 2. Case Presentation

**Figure 1:**
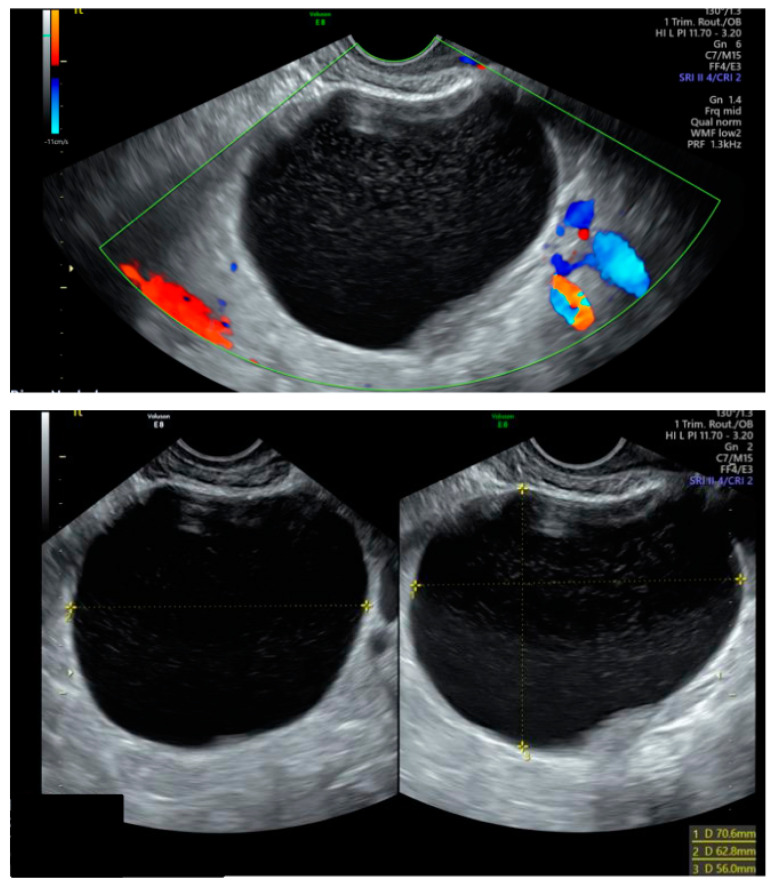
Transvaginal appearance of the left ovarian cyst at 5 weeks of gestation.

Our case involves a 32-year-old primigravida of Asian origin, who was classified as low-risk during her initial antenatal booking, with normal first-trimester screening results, and presented at 5 weeks’ gestation with lower abdominal pain and light vaginal bleeding. A transvaginal ultrasound confirmed a viable intrauterine pregnancy and identified a 71 × 63 × 56 mm left ovarian cyst with a ground glass appearance, suggestive of an endometriotic cyst (Figure 1). The subsequent anomaly scan at 20 weeks noted a low-lying placenta, fibroids, and an ovarian cyst that remained unchanged in size without exhibiting concerning ultrasonographic features.

At 24 weeks and 3 days of gestation, she presented with abdominal pain and a sensation of abdominal heaviness and bloating. Her abdominal examination was normal, and she was managed conservatively. Growth scans at 28 and 32 weeks indicated normal foetal growth, amniotic fluid volume, and Doppler signals. An anterior intramural fibroid measuring 44 × 21 × 26 mm was identified, and the left ovarian cyst, now reported as 70 × 55 × 60 mm, exhibited internal echoes and an avascular solid component, representing a follicular cyst with debris or a cystic neoplasm at 32 weeks. Due to the complex nature of the cyst, tumour markers were measured, and a plan for a multidisciplinary team (MDT) discussion was made. Her tumour markers were normal (CEA < 2, CA-19-9 < 2, and CA-125 14). The MDT meeting concluded that the cyst appeared simple with high gain and recommended a repeat ultrasound to rule out any solid areas. The patient’s symptoms improved without the need for further surgical intervention at that stage.

At 36 weeks and 3 days, the patient presented acutely with left lower abdominal pain and vomiting. She denied any vaginal bleeding or urinary symptoms, reported regular bowel movements, and had no constipation or low urinary tract symptoms. Her observations were stable, and she appeared clinically well. Examination revealed slight tenderness on the left side of the abdomen, but no guarding was elicited. The uterus was soft and relaxed. Foetal movements were normal, as was foetal monitoring.

Her admission blood test results were as follows: Hb 121 g/L, white cell count (WCC) 10.0 × 10^9^/L, platelets 270 × 10^9^/L, neutrophils 9.1 × 10^9^/L, and C-reactive protein (CRP) 30 mg/L. Given the clinical picture and ultrasound findings for the ovarian cyst, there was a discussion about preterm delivery versus conservative management and delivery by 38 weeks unless ovarian torsion became a concern.

**Figure 2:**
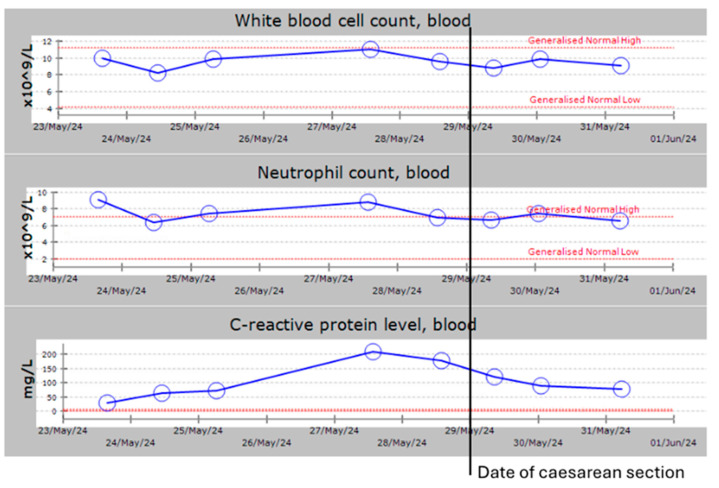
The trend regarding white blood cell count, neutrophil count, and C-reactive protein levels in the antenatal and postnatal periods.

As her pain intermittently resolved with regular analgesia and she did not exhibit clinical peritonitis, she was initially managed conservatively. During three days of inpatient admission, her CRP rose to 209 from 30, while her WCC remained normal (Figure 2). Despite no pyrexia, intravenous antibiotics (cefuroxime and metronidazole) were started due to the raised inflammatory markers.

**Figure 3:**
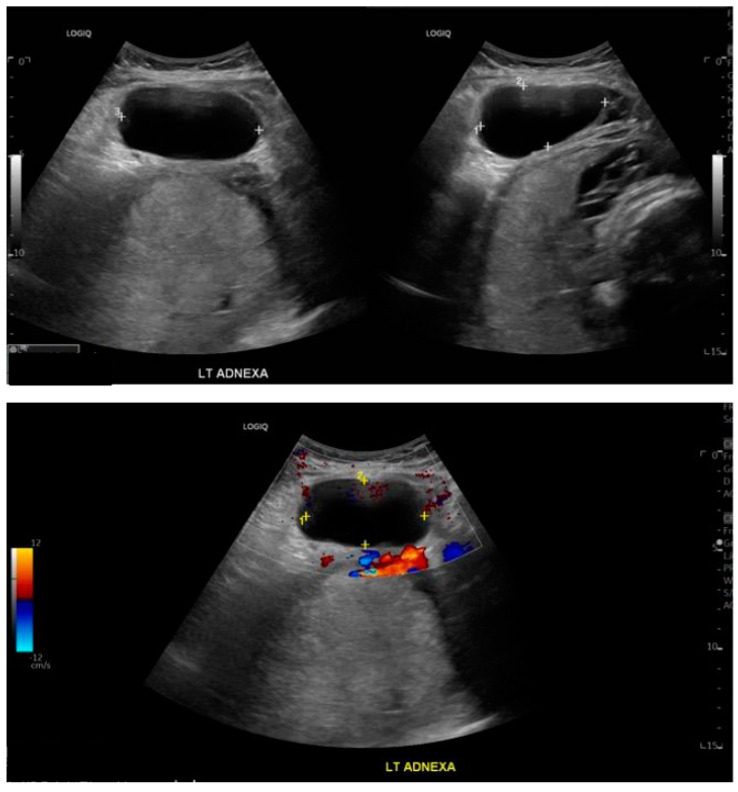
Transabdominal appearance of the left ovarian cyst at 36 + 2 weeks of gestation.

Repeated transabdominal and transvaginal ultrasounds, performed by an experienced sonographer, showed that the known left adnexal cyst remained the same in size and appearance at 63 × 33 × 69 mm, with no features of torsion noted (Figure 3). Specifically, ultrasonographic signs indicative of ovarian torsion, such as the ‘whirlpool sign’, ovarian stromal oedema, absence of detectable Doppler signals in the ovary, and the presence of free fluid in the pelvis, were not observed. There was a discussion about performing an MRI to assess the ovarian cyst, as ultrasound findings were inconclusive. However, due to the rapid change in the clinical picture, an MRI was not performed.

At 37 weeks and 1 day, her pain increased and was associated with vomiting. On abdominal examination, she had generalised tenderness and guarding in the left iliac fossa. Given the development of acute abdomen associated with an uptrending CRP and suspicion of ovarian torsion, the decision to proceed with a caesarean section to deliver the foetus, followed by surgical management of the ovarian torsion, was made and discussed with the patient.

**Figure 4:**
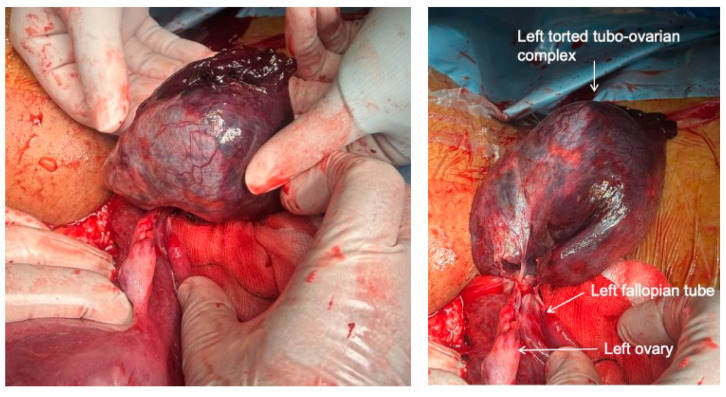
Intra-operative findings during caesarean section.

Intraoperatively, the infant was delivered in good condition and had good APGAR scores. The uterus was closed in double layers, and haemostasis was achieved. The left ovarian cyst was found to be diffusely necrotic, twisted several times around its supporting ligaments, and appeared blue/–black, forming a complex with the lateral third of the fallopian tube (Figure 4). The non-viable left tubo-ovarian complex, including the twisted ovarian cyst, was removed, while part of the ovary with viable macroscopic tissue characteristics was preserved, maintaining blood supply through branches of the utero-ovarian ligament. Additionally, a 3 × 4 cm sub-serosal sessile fibroid and a 1 cm fibroid on the anterior surface of the uterus were identified, both appearing normal without signs of degeneration. The estimated blood loss was 400 mL, and the postoperative period was uneventful. Histopathological examination revealed a 6 cm long fallopian tube with internal haemorrhage and necrosis, along with a benign serous cystadenoma of ovarian origin exhibiting internal haemorrhage and necrosis.

## 3. Discussion

In this study, we present the case of a 32-year-old primigravida who experienced abdominal pain at 36 weeks and 3 days of gestation and developed an acute abdomen secondary to tubo-ovarian complex torsion. The patient had an emergency caesarean section at 37 weeks and 1 day of gestation with simultaneous excision of the left twisted tubo-ovarian complex with preservation of the macroscopic viable ovarian tissue.

Ovarian torsion is defined as the rotation of the ovarian pedicle around its own axis, leading to ischaemia and ovarian necrosis. It is a rare gynaecological cause of acute lower abdominal pain, occurring in approximately 1–5 in 10,000 pregnancies [5]. Incidence is increased in pregnancy due to upward displacement of ovaries by the uterus. It can manifest in all trimesters; however, a systematic review of the literature found that the incidence of adnexal torsion in pregnancy is lowest in the third trimester [2]. Two-thirds of the adnexal torsion occurs on the right side, as the left sigmoid colon limits movement in the left ovarian pedicle [6]. The symptoms of torsion are usually nonspecific and mimic other common obstetric conditions, such as preterm labour, placental abruption, and non-gynaecological conditions such as gastroenteritis, renal colic, and appendicitis [7]. Common symptoms include acute-onset abdominal pain, often accompanied by nausea, vomiting, low-grade fever, or leucocytosis.

Adnexal masses are one of the most common complications during pregnancy, with an occurrence rate ranging from 1 to 5% [8]. The increasing use of first-trimester ultrasound has led to an increased diagnosis of adnexal masses in asymptomatic pregnant women. Differential diagnoses of ovarian masses in pregnancy include functional cysts; benign ovarian cysts such as cystadenomas; endometriomas; mature cystic teratomas; dermoid cysts; and malignant ovarian neoplasms such as germ cell tumours, cystadenocarcinomas, and sex cord–stromal tumours. Although most of the ovarian cysts diagnosed in pregnancy are benign and asymptomatic, approximately 2% of adnexal masses in pregnancy are malignant, and some may warrant surgical intervention because of size, symptoms, risk of torsion, or suspicion of malignancy [9].

In this case, the clinical suspicion for torsion was high, as the patient had a known 7 cm left-sided adnexal cyst. Although her observations remained stable with no demonstrated features of torsion on ultrasound scan, the rise in inflammatory markers was noticed, and her symptoms exacerbated during the course of admission. Although ultrasound is deemed as the mainstay diagnostic tool for evaluating adnexal mass in pregnancy, its diagnostic accuracy is limited owing to the gravid uterus displacing the ovary from its normal location. Nevertheless, it is recommended as the first-line diagnostic tool to comment on any possible enlarged heterogenous adnexal masses, as well as ovarian vessel flow, using colour Doppler or signs of excess pelvic fluid [1]. Magnetic resonance imaging (MRI) can be more helpful in cases where ultrasound is not sufficient for evaluating the adnexa.

Management of adnexal masses in pregnancy varies depending on the gestational age, the size of the cysts, and the clinical presentation. Ovarian cysts less than 6 cm in diameter and appearing benign on ultrasound are generally managed conservatively with serial ultrasound surveillance, as they usually resolve spontaneously [4]. Cysts exceeding 10 cm in size are usually resected due to the increased risk of malignancy, rupture, or torsion, with both open and laparoscopic approaches being described in the literature [10]. Cysts that are >6 cm, complex, and multiloculated are more likely to persist and are prone to torsion, as described in our case. Cysts more than 10 cm are usually resected due to increased risk of malignancy, rupture, or torsion. The management of cysts between 5 and 10 cm is controversial. If the cysts show signs of septation, nodules, papillary excrescences, or solid components, then resection is recommended [11].

Ovarian cysts during pregnancy may be managed conservatively if diagnosed in the first trimester, depending on size, ultrasonographic features, and the clinical status of the patient. The timing of intervention presents a significant challenge, with the ideal window for surgical intervention generally considered to be between 16 and 28 weeks of gestation. In our case, surgical intervention was performed at term due to clinical uncertainty regarding the diagnosis and to optimise neonatal outcomes. However, it is important to note that earlier intervention might have prevented the removal of ovarian tissue as part of the torted tubo-ovarian complex. The balance between the risks of surgical complications and the maternal and neonatal outcomes should always be carefully considered, ideally with the involvement of a multidisciplinary team.

Treatment is limited to surgery, either by laparoscopy or laparotomy. Laparoscopy has been increasingly utilised for both the diagnosis and treatment of adnexal torsion in early pregnancy and has shown some benefits, such as reduced hospital length of stay and postoperative pain, and adverse foetal outcomes, such as foetal loss, premature delivery, and stillbirth [12,13]. However, laparoscopy may be challenging in advanced gestation due to the limited working space and the risk of injuring the gravid uterus. In this case, laparotomy was a safer approach, as it achieved delivery of the foetus concurrently. Cystectomy with detorsion is usually the most recommended approach in cases of ovarian torsion, even if the ovaries are ischaemic. However, if there is irreversible damage to the ovary, salpingo-oopherectomy may be performed [14].

## 4. Conclusions

Ovarian torsion in the third trimester of pregnancy is a rare but serious condition that requires a high index of clinical suspicion for diagnosis. Prompt imaging and surgical intervention are critical to prevent adverse outcomes for both the mother and foetus. This case highlights the challenges of obtaining ultrasound images indicative of torsion in advanced pregnancy. It underscores the importance of strong clinical suspicion for diagnosing torsion. The combined approach of caesarean section, partial salpingo-oophorectomy, and cystectomy at 37 weeks allowed for the safe delivery of the foetus, the removal of the twisted tubo-ovarian complex mass, and the preservation of residual normal ovarian tissue, demonstrating the efficacy of timely surgical intervention. This case underscores the importance of multidisciplinary care and the need for further research into the management of ovarian torsion in late pregnancy.

## Data Availability

The original contributions presented in the study are included in the article. Further inquiries can be directed to the corresponding author.
